# The three stages of building and testing mid-level theories in a realist RCT: a theoretical and methodological case-example

**DOI:** 10.1186/s13063-015-0980-y

**Published:** 2015-10-15

**Authors:** Farah Jamal, Adam Fletcher, Nichola Shackleton, Diana Elbourne, Russell Viner, Chris Bonell

**Affiliations:** Department of Social Science, UCL Institute of Education, 18 Woburn Square, London, WC1H 0NR UK; School of Social Sciences, Cardiff University, 1-3 Museum Place, Cardiff, CF10 3BD UK; UCL Institute of Child Health, 30 Guilford Street, London, WC1N 1EH UK; Medical Statistics Department, London School of Hygiene and Tropical Medicine, Keppel Street, London, WC1E 7HT UK

**Keywords:** Realist, Randomised controlled trials, Complex interventions, Social experiments, Generalisability, Social epidemiology, Schools

## Abstract

**Background:**

Randomised controlled trials (RCTs) of social interventions are often criticised as failing to open the ‘black box’ whereby they only address questions about ‘what works’ without explaining the underlying processes of implementation and mechanisms of action, and how these vary by contextual characteristics of person and place. Realist RCTs are proposed as an approach to evaluation science that addresses these gaps while preserving the strengths of RCTs in providing evidence with strong internal validity in estimating effects.

**Methods:**

In the context of growing interest in designing and conducting realist trials, there is an urgent need to offer a worked example to provide guidance on how such an approach might be practically taken forward. The aim of this paper is to outline a three-staged theoretical and methodological process of undertaking a realist RCT using the example of the evaluation of a whole-school restorative intervention aiming to reduce aggression and bullying in English secondary schools.

**Discussion:**

First, informed by the findings of our initial pilot trial and sociological theory, we elaborate our theory of change and specific a priori hypotheses about how intervention mechanisms interact with context to produce outcomes. Second, we describe how we will use emerging findings from the integral process evaluation within the RCT to refine, and add to, these a priori hypotheses before the collection of quantitative, follow-up data. Third, we will test our hypotheses using a combination of process and outcome data via quantitative analyses of effect mediation (examining mechanisms) and moderation (examining contextual contingencies). The results are then used to refine and further develop the theory of change.

**Conclusion:**

The aim of the realist RCT approach is thus not merely to assess whether the intervention is effective or not, but to develop empirically informed mid-range theory through a three-stage process. There are important implications for those involved with reporting and reviewing RCTs, including the use of new, iterative protocols.

**Trial registration:**

Current Controlled Trials ISRCTN10751359 (Registered 11 March 2014)

## Background

Most major public health challenges today are complex and require interventions to address multiple determinants at the individual and wider socio-ecological levels [1, 2]. Appropriately designed and conducted randomised controlled trials (RCTs) represent the most internally valid means of estimating the effects of complex interventions to determine if particular interventions are effective or not overall. However, RCTs of such interventions are often criticised as failing to open the ‘black box’ – that is, they examine quite crude questions about ‘what works’ without explaining the underlying processes of implementation and mechanisms of action, and how these vary by contextual characteristics of person and place.

While some authors have argued that small but important treatment effects will be fairly generalisable across contexts [3], this is only in relation to pharmaceutical trials. Public health interventions on the other hand are far from straightforwardly generalisable as they involve the complex interplay of social structure and agency, shaping intervention implementation and how people respond to it, which will vary markedly in different national and local contexts. For this reason, they can be conceptualised as ‘interruptions’ to complex social systems [4]. If the results of public health evaluations are to be useful in informing decisions of whether interventions are to be deployed elsewhere, greater consideration of the external validity of evaluation results is needed. Integral to this is a consideration of not only whether interventions can feasibly be delivered in a new setting, but also a clear understanding of intervention implementation and causal mechanisms and how each of these might vary with context [5].

### Realist RCTs

Realist RCTs have been proposed as an approach to evaluation science that addresses these gaps while preserving the strengths of RCTs in providing evidence with strong internal validity in estimating effects [6, 7]. Realist evaluators have viewed interventions as ‘working’ by introducing *mechanisms* that interact with features of their *context* to produce *outcomes*, and they ask and answer questions not only about what works at an aggregate level but also w*hat works for whom and under what circumstances* [8].

A key aspect of realist evaluation is to understand how the intervention works via anticipating the diversity of potential intervention mechanisms, presenting this in a theory of change and then assessing empirically whether and how these mechanisms are ‘enabled’ or ‘disabled’ in the varying contexts in which the intervention is delivered, and how this may vary for different groups of people. Context refers to the pre-existing set of social situations, norms, values and inter-relationships (e.g. organisational structure, geographic location, demographics of participants) within which an intervention is implemented. Thus, the evaluator needs to hypothesise and test how the intervention theory of change interacts with context to enable (or disable) implementation, causal mechanisms and, ultimately, outcomes. Pawson and Tilley [8] describe this process of hypothesis building as developing ‘context-mechanism-outcome (CMO) configurations’. Traditionally, realist evaluators examine these hypotheses using observational data, rejecting the use of random allocation and control groups as embodying a positivist epistemology anathema to their own realist orientation.

Proponents of realist RCTs accept the argument that traditional evaluations have been too focused on questions of overall effects, as well as the necessity of theorising and empirically evaluating how intervention mechanisms interact with context to generate outcomes. However, advocates of realist RCTs also recognise that a randomly allocated comparison group offers the least biased means of determining the direction and size of any intervention effects and how these are moderated by context, pointing out that observational studies are often stymied by confounding by baseline non-equivalence. Realist RCTs are, therefore, a design that allow evaluations to be focused on refining generalisable intervention theory, as well as accrediting particular interventions as effective or not, as both questions can be examined within modified RCT designs [6, 7, 9]. Proponents of the realist RCT approach reject the notion that the RCT method is necessarily positivist, citing literature that research cannot in practice be reduced to discrete, incommensurate paradigms and that methods do not necessarily imply epistemologies [10]. Realist RCTs are also distinct from realist evaluations nested within RCTs but which proceed in parallel without using randomised comparisons as an analytical resource [11].

While some originators of realist evaluation reject the argument that realist analysis can draw on experimental data [12], new Medical Research Council (MRC) guidance on process evaluation [9, 13] has recognised the value of realist RCTs in asking a range of questions about implementation, context, mechanisms, outcomes and normalisation. However, the realist RCT approach has so far only been described in theory [6, 10] and no detailed examples have been offered. In the context of growing interest in designing and conducting realist RCTs, there is an urgent need to provide practical guidance on how such an approach might be taken forward.

### Aims of this paper

This paper provides a worked example of the theoretical and methodological process of undertaking a realist RCT drawing on the evaluation of the Learning Together (LT) intervention. This is a 3-year ‘phase III’ [2] cluster RCT of effectiveness across 40 schools (20 in the intervention group and 20 in the comparison group) in England to evaluate a whole-school restorative approach to reduce student bullying and aggression [14]. Building on an earlier pilot trial undertaken in 8 schools over 1 school year (2011–2012) [15], this phase III RCT commenced in September 2014 and is currently underway. A protocol of the RCT is already published [14].

Using this example, this paper describes the stages via which a realist RCT develops and tests hypotheses about how the intervention mechanisms interact with diverse contexts to generate outcomes. The published trial protocol does not address these issues and is focused on the methods for assessing overall effects and intervention fidelity. This paper does not deviate from this protocol [14] but rather expands on it to provide a worked example for researchers, practitioners and policymakers seeking to design realist RCTs, although it does not provide study results as these are not yet available. We also reflect on important implications for those involved with reporting and reviewing realist RCTs, including recommendations for new, iterative protocols that could be updated online at the multiple stages of building and testing intervention theory.

### The Learning Together intervention

Learning Together (LT) is a whole-school restorative approach to reducing aggression and bullying in English secondary schools (students aged 11–16). It combines pre-specified processes (a survey of student needs; an action group comprising a selection of staff and students; training for staff in using restorative practices to prevent and address misbehaviour) and outputs (review and revision of school rules and policies; student social and emotional skills curriculum) with the capacity for tailoring some actions locally. The action group is tasked with reviewing data from a survey about student aggression and bullying as well as determinants of these behaviours such as connection to the school, engagement with learning and quality of relationships (the survey of student needs). Based on this review, the group determines priorities for action, some of which are pre-specified and consistently undertaken across all schools (e.g. revisions to school rules and policies) while others are locally decided by the staff and students [15]. The school action group is supported by an external intervention facilitator. A detailed description of the LT intervention is reported elsewhere [14, 15]. The intervention has been developed using a phased approach as recommended by the MRC [2]. It was piloted in 4 schools in 2011–2012 within a pilot RCT primarily examining feasibility of the intervention and trial methods [15].

## Methods

**Fig. 1:**
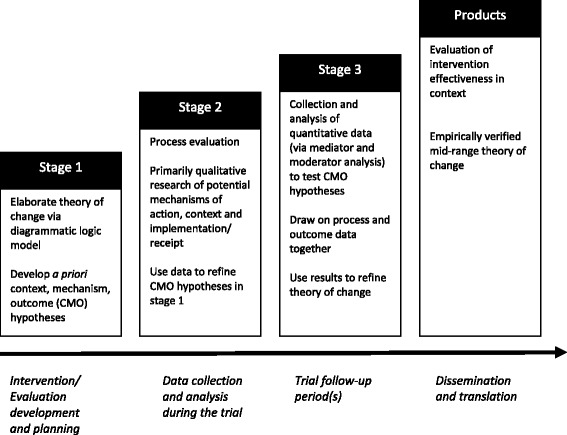
Summary of staged framework for a realist randomised controlled trial (RCT)

Our realist RCT of the Learning Together intervention proceeds via a number of stages, only the first of which has so far been completed. A summary of the staged theoretical and methodological framework for realist RCTs is provided in Fig. 1, and each stage is outlined in detail below. Our first stage consisted of developing various *a priori* hypotheses about how intervention *mechanisms* might interact with *context* differentially to produce *outcomes* (CMO configurations). We did this at the start of the current phase III trial. The CMO hypotheses were informed by the findings of the prior pilot trial and an earlier feasibility study [15, 16], as well as by the sociological theory that informed the original intervention logic model and design [17] and empirical evidence regarding the processes of school effects on health [10, 18]. The second stage will involve refining or augmenting this limited list of a priori hypotheses prior to the collection of quantitative, follow-up data. This will be informed by the findings which will emerge from the process evaluation which is integral to the current phase III RCT. In the third stage, we will test our hypotheses using a combination of process and outcome data from the phase III trial: for example, to examine moderation and mediation. Our aim is thus not merely to assess whether LT is an effective intervention or not, but to develop empirically informed mid-range theory [6, 19] (i.e. theory about empirical phenomena that can be verified with data) about school processes, how these may be modified to reduce aggression and bullying, and how this is shaped by context.

### Stage 1: pre-hypothesised theory of change and CMO configurations

**Fig. 2:**
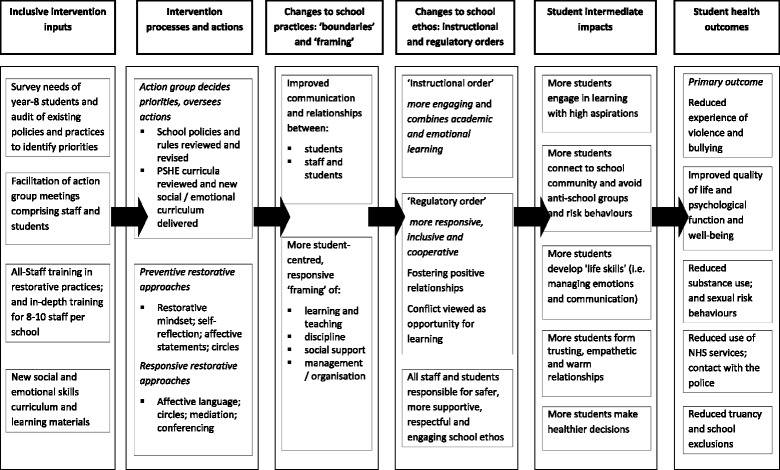
Diagrammatic logic model of the Learning Together intervention

Our original theory of change drew predominantly upon sociological theory, focusing on system-level change. Before the earlier pilot trial, we described this initial theory of change using a diagrammatic logic model (Fig. 2). Informed by Markham and Aveyard’s [17] theory of human functioning and school organisation, it started from the theoretical position that schools have a wide-ranging influence on students’ attitudes and action. Students’ capacities for practical reasoning (thinking) and affiliation (forming relationships), which are essential to their choosing to avoid bullying, aggression and other risk behaviours, are theorised as being facilitated by their being committed to a school’s ‘instructional’ and ‘regulatory’ orders. Based on Bernstein’s work [20], the instructional order refers to school processes of enabling student learning. The regulatory order focuses on how the school inculcates student values and sense of belonging.

The theory suggests that schools build student commitment to these instructional and regulatory orders by modifying ‘boundaries’ and ‘framing’ [17]. It is theorised that if schools reduce barriers between the school and the communities they serve, between students and teachers, between student groups, and between academic subjects, and if they increase the extent to which provision is framed in terms of the needs and preferences of students, then more students will become committed to the instructional and regulatory orders of the school [17] and will develop the practical reasoning and affiliations necessary to avoid risk behaviours.

The LT intervention aims to reduce bullying and aggression via boundary erosion and increasing student-centred ‘framing’ of school provision to promote students’ commitment to the school instructional and regulatory orders. These mechanisms encourage students to make healthier choices and the intervention is a way to trigger these mechanisms. The original theory of change was presented in a diagrammatic logic model of intervention processes, mechanisms and outcomes at the end of the pilot trial (Fig. 2). The model at this stage was linear, specifying hypothesised causal pathways that would lead to intended health benefits in ideal circumstances. This linearity reflected our initial lack of engagement with realist evaluation as well as our desire to present a relatively clear two-dimensional diagrammatic summary of the intervention to schools. The linear logic model was, however, a useful starting point in setting out the main mechanisms to be examined in the current realist RCT, which we hope will occur in most schools (boundary erosion, student centring and student commitment to instructional and regulatory orders) in order to enable intervention benefits to be realised.

#### Pre-hypothesised intervention mechanisms

To examine empirically whether this is the case we will examine various mediation hypotheses suggested by this logic model and the intervention theory underlying it:

- ▪ Hypothesis 1: LT schools will report reduced student-student, student-staff and academic-broader learning boundaries and increase student-centred framing at follow-up.
- ▪ Hypothesis 2: LT schools will report higher rates of student commitment to the schools’ instructional and regulatory orders by follow-up.
- ▪ Hypothesis 3: LT schools will report higher rates of student life skills and positive, trusting and empathetic relationships and lower rates of student involvement in anti-school peer groups by follow-up.
- ▪ Hypothesis 4: intervention effects on primary and secondary trial outcomes will be mediated by these reductions in boundaries, increases in student-centred framing, student commitment, skills and relationships, and decrease in involvement in anti-school peer groups.

#### Pre-hypothesised contextual barriers and facilitators

The logic model as developed at the end of the pilot trial (presented in Fig. 2) was also limited by not engaging with how contextual factors might modify intervention implementation and mechanisms. We will develop hypotheses about this in the course of the evaluation (see stage 2, below) but we started with a number of a priori hypotheses about how context might moderate intervention mechanisms and outcomes, informed by literature on the sociology of education, youth and health and previous similar interventions (cited below).

- ▪ Hypothesis 1: the intervention will be more acceptable and be implemented with better fidelity when it is line with existing school institutional approaches and teacher practices which aim to erode the boundaries which the intervention is addressing [18, 21].
- ▪ Hypothesis 2: the intervention will be implemented with better fidelity when the school has the capacity to implement it properly, in terms of: the action team being chaired or otherwise led by a person with real authority in the school; the action team involving other individuals, which means it is taken seriously both by staff and students; the action team being formally linked in to the school decision making structures (e.g. through involvement of a senior leader); and the school being a generally functional institution: e.g. stable staffing, not in crisis with respect to targets and inspections [15, 18, 21].
- ▪ Hypothesis 3: the intervention will be implemented with better fidelity in schools that include students with varying degrees of educational engagement in its activities (e.g. action groups), including students who have a history of, or are considered likely to be involved in bullying behaviours [15, 16].
- ▪ Hypothesis 4: drawing on evidence from cross-sectional studies from the United States (US) [22, 23], we hypothesise that schools will implement restorative approaches with less fidelity of function in schools with higher numbers of African Caribbean or minority ethnic students. The hypothesis suggests that schools may be unconsciously prejudiced in their practice. A theoretical explanation proposed is that school institutions (similar to criminal justice systems) see a high ratio of ethnic minorities as a perceived threat and intensify punitiveness. These processes may play out differently in the UK which is a different social and historical context from the US.
- ▪ Hypothesis 5: the intervention will be more effective in schools with more students of low socio-economic status (SES) backgrounds since eroding boundaries is hypothesised as more important for these students [17].

Regarding gender, a similar whole-school intervention addressing bullying and aggression in the US [24] reported a range of benefits for boys but not girls. However, in the absence of a process evaluation, the reasons for these differential effects are unclear. Therefore, before developing hypotheses regarding the gendered nature of effects, we will examine emerging data from our process evaluation with a focus on gender to hypothesise at stage 2 (below) whether and how the intervention may be implemented for and received by girls and boys.

This development of CMO configurations meant that our theory of change moved from being simplistic, linear and acontextual to being realist and contextual. However, we retained our original, linear logic model since incorporating all these CMO configurations into a single diagram would have involved mapping out a complex array of processes in a three-dimensional model, which would have lost any clarity. The logic model, though acontextual, nonetheless suggests how the intervention might proceed under ideal conditions and is also useful to communicate the basic intervention theory with schools, young people, parents and other groups.

### Stage 2: empirical process evaluation to refine CMO hypotheses

In this stage, which is ongoing, we will refine and augment the theory and hypotheses developed in stage 1 by drawing on empirical evidence. In our phase III trial’s integral process evaluation, we are collecting data on intervention implementation, receipt, acceptability and normalisation (i.e. sustainability), as well as mechanisms and context. In traditional trials, process evaluation is used to examine intervention fidelity so that it can be determined whether any limitations in effectiveness reflect a true lack of effectiveness of the intervention model or merely a failure of implementation of that model. However, our process evaluation will go beyond this to explore intervention mechanisms and how these interact with context to generate outcomes (or fail to do so).

Data are being collected via:

- ▪ diaries completed by intervention deliverers (trainers and external facilitators working in schools to establish and support action groups);
- ▪ researcher observations;
- ▪ interviews with school staff, students and intervention deliverers;
- ▪ surveys that monitor satisfaction and implementation of core components of the intervention; and
- ▪ in-depth case studies involving participant observation, focus groups and interviews in a selection of intervention schools.

Qualitative research captures a sense of research participants’ own meanings, their sense of agency and how this inter-relates with the social structure of intervention context. It can thus suggest hypotheses about the complex mechanisms by which our intervention might work, including those not originally anticipated by us in stage 1 and those issues that were under-theorised at stage 1 (e.g. gender). Qualitative data may provide important insight into contexts and unintended pathways that can then be tested via quantitative mediation and moderation analyses. While qualitative research can also be conducted after quantitative analyses to try to explain unexpected findings, this is less than ideal since it may mean that quantitative analyses are insufficiently focused on testing hypotheses (and so vulnerable to charges of ‘data dredging’) and qualitative analyses may be biased towards searches for data that confirm quantitative findings. Thus, during the course of the trial, the qualitative data described above will be analysed using framework analysis and grounded theory methods [25] to refine our theory and CMO hypotheses prior to quantitative testing in stage 3.

### Stage 3: testing mid-level CMO theories

In this stage, we will test hypotheses derived in stages 1 and 2 via quantitative analyses of effect mediation (to examine mechanisms) and moderation (to examine contextual contingencies). There are concerns among trialists that within trials multiple analyses can lead to false positive results [26]. However, our approach of grounding these in hypothesis testing minimises these risks and ensures transparency. The use of prior theory and empirical evidence derived from stages 1 and 2 means that the quantitative testing described below will be limited in number and focused on promising hypotheses rather than being unfocused ‘fishing expeditions’ likely to produce as many false as real positive results [27–29]. Combining process and outcome data enables us to develop empirically informed mid-range theory [6, 19] about school processes and how these may be modified by the intervention, and the extent to which LT may be transferable to a range of population and contexts. Causal mediation analysis helps to identify process or mediating variables that lie in the causal pathways between the treatment and the outcome [30, 31]. Mediators are post-baseline measures of interim effects which may or may not account for intervention effects on end-outcomes. In our phase III trial, mediation analysis will assess whether intervention effects on aggression and bullying might be accounted for by intermediate effects on: boundaries between and among student and staff and between students’ academic and broader learning boundaries; the student-centred framing of provision; student commitment to schools’ instructional and regulatory orders; student life skills and affiliation; and student involvement in anti-school peer groups. For example, we will examine if boundaries between staff and students mediate any effects of the LT intervention on bullying and aggression in schools. Within the context of evaluating social interventions such as this, measuring underlying change mechanisms (i.e. mediators) as well as outcomes provides information about which mechanisms are critical for influencing outcomes. This information will enable evaluators conducting realist RCTs to refine the theory of change at this stage to focus on effective components of interventions, specify causal pathways and remove ineffective components and insignificant mechanisms responsible for change. Table 1 illustrates the variables and scales used to test mediators in the LT trial. These map directly on to the pathways and constructs presented in the logic model in Fig. 2. Ultimately the analysis will assess whether these appear to mediate intervention beneficial effects on primary and secondary trial outcomes.

**Table 1:** School-level and individual-level pre-hypothesised mediators of Learning Together

School-level variables		
Learning Together theory of change constructs		Selected items from the following originator scales
Aggregate student perception of staff-student boundaries		Beyond Blue School Climate Questionnaire^a^
Aggregate student perception of student-centred framing of school		Beyond Blue School Climate Questionnaire^a^
Aggregate staff perception of staff-student boundaries	Teacher authority or teacher-student collaboration	School policies and practices survey^b^
	Teacher support for students across school or restriction to classroom	School policies and practices survey^b^
Aggregate staff perception of academic/broader development boundaries		School policies and practices survey^b^
Aggregate staff perception of student-student boundaries	Dividing up or bringing together students (learning)	School policies and practices survey^b^
	Dividing up or bringing together students (discipline and pastoral)	School policies and practices survey^b^
Individual level variables		
Learning Together theory of change constructs		Originator scales
Student commitment to school regulatory order		Beyond Blue School Climate Questionnaire^a^
Student commitment to school instructional order		Beyond Blue School Climate Questionnaire^a^
Student capacity for affiliation		The Strengths and Difficulties Questionnaire^c^
Student capacity for practical reasoning	Empathy with others	The Strengths and Difficulties Questionnaire^c^
	Ability to manage own emotions	The Strengths and Difficulties Questionnaire^c^
		Short Warwick-Edinburgh Mental Well-Being Scale^d^
	Ability to manage/not manage conflict	The Strengths and Difficulties Questionnaire^c^
Involvement with anti-school peer groups		ESYTC Self-Reported Delinquency^d^
		Young People’s Development Programme (YPDP) evaluation measure^e^

^a^Sawyer et al., 2010 [40]

^b^Newly developed scale by the Learning Together project team

^c^Goodman, 1997 [41]

^d^Clarke et al., 2011 [42]

^e^Wiggins et al., 2009 [43]

Whereas mediators establish ‘how’ or ‘why’ one variable predicts or causes a change in an outcome variable, moderators address ‘when’ or ‘for whom’ a predictor is more strongly related to an outcome [31]. Operationally, a moderator is a baseline variable that alters the direction or strength of the relationship between a predictor and an outcome [31]. This allows evaluators to investigate not only the general effectiveness of an intervention, but which interventions work best for which people and in which settings [31].

Informed by previous whole school interventions [21] and the pilot trial [16] in LT, we hypothesise that schools already aiming to erode staff-student boundaries before the intervention commences are more likely to implement school level actions via the school action group meetings to change school boundaries and thus ultimately report lower levels of bullying and aggression at follow-up. We also hypothesise that schools with higher levels of students of low socio-economic status will report lower levels of bullying and aggression at follow-up. Thus, schools’ priorities (assessed via baseline interviews with school staff) and students’ socio-economic status are both introduced as moderators of the relationship between the intervention and levels of bullying and aggressive behaviour. These interaction effects (i.e. moderators) are important because they allow evaluators to assess whether the intervention is inappropriate at a particular time and context or even potentially harmful. In our trial, we will examine various school-level and individual-level characteristics that moderate implementation and mechanisms. Table 2 summaries the data sources used for this examination. With only 20 intervention schools we are unlikely to be able to test statistically whether implementation and effectiveness is significantly better in the types of school contexts set out in the above CMO configurations, so most of these data and analyses will be qualitative to support hypothesis building rather than testing. Given some of the constraints on testing CMO hypothesis, we allow for the possibility that the results of these analyses may be indeterminate or negative with the theory of change.

**Table 2:** Exploring pre-hypothesised moderators of Learning Together (LT)

Context (moderator)	Enables or constraints mechanisms:	Outcome	Data collection
School already aiming to erode staff-student boundaries	Implementation of actions to change school boundaries	Hypothesised intermediate outcomes: Students engage in learning with high aspirations; more students connect to school and avoid risk; more students develop life skills; more students form trusting relations	School policy and practice survey. Fidelity and staff acceptability data
School already aiming to erode staff-staff boundaries	Implementation of actions to change school boundaries		School policy and practice survey. Fidelity and staff acceptability data
School already aiming to erode boundaries between academic and broader development	Implementation of actions to change school boundaries	Hypothesised primary and secondary outcomes: Reduced bullying and aggression; improved quality of live and emotional and mental health; reduced substance use and sexual risk; reduced truancy and school exclusions	School policy and practice survey. Fidelity and staff acceptability data
School organisational capacity; staff turnover; stability	Implementation of any intervention activities.		Routine monitoring data; School policy and practice survey. Fidelity data
Inclusion of students with varying degrees of educational engagement in LT activities including bullies	Implementation of actions to change school boundaries.		Facilitator diary forms; interviews with staff/student action group members AGM. Fidelity data
	Increased commitment of disengaged students.		
Ethnic composition of students	Implementation of restorative approaches.		Student survey. Fidelity of restorative approaches data
Socio-economic status of students	Increased commitment of critical mass of disengaged students.		Routinely collected data; student survey

### Ethical issues

The Learning Together trial, which is used as a worked example in this manuscript has been approved by the Institute of Education Research Ethics Committee (18 November 2013 ref. FCL 566) and the University College London Research Ethics Committee (30 January 2014, Project ID: 5248/001).

## Discussion

This paper provides the first guidance on the theoretical and methodological process of undertaking a realist RCT using the example of the evaluation of the Learning Together intervention. It outlines a three-staged process aimed at developing, testing and refining hypotheses in order to arrive at an empirically informed mid-range theory of change. Thus, the main products of a realist RCT are both an evaluation of particular intervention effectiveness in a particular context as well as a nuanced mid-range theory of change (Fig. 1), rather than outputs which merely accredit an intervention as effective or not. When simply the empirical fact of ‘what works’ is known, an intervention can only be reproduced in the hope it will have expected effects [32] or it falls into the ether of evidence and has little or no influence on population health. When we understand what works, for whom and under what circumstances, interventions can be translated and adapted to varying populations and contexts.

We recognise that many existing trials are powered on the basis of overall intervention effects on a primary outcome so that mediator and moderator analyses such as those described above may be underpowered. However, we would make the point that trials already commonly include mediation and moderation analyses; we simply recommend that such analyses be more focused on testing hypotheses about how intervention mechanisms interact with context to produce outcomes, and that such hypotheses be more informed by intervention theory and preliminary analysis of data from process evaluations. One such example is the Welsh National Exercise Referral Scheme (NERS) policy trial that built on a formative process evaluation to develop an intervention logic model and hypothesise potential mechanisms of action and moderators [33]. Mediation analyses of trial data suggested that changes in physical activity can be partly explained by changes in autonomous motivation and social support [34], which should, therefore, be central to planning future exercise referral programmes. Moderation analyses were also able to examine how effects varied by subgroup and found that the programme did *not* increase physical activity for those patients referred for mental health reasons but did for those referred on the basis of coronary heart disease risk [33]. Future trials may even be powered specifically to examine mediation and moderation rather than merely overall effects.

Furthermore, where multiple trials examine similar CMO configurations, meta-analyses can examine mediation and moderation with more power. For example, a review and meta-analysis of criminal justice interventions by Lipsey [35] examined how the site of delivery moderated effectiveness. Finally, while individual moderation and mediation analyses from most single trials may be underpowered to perform definitive analyses, they might nonetheless still be useful in refining hypotheses and intervention theories.

We recommend that realist RCTS are still guided by a priori published protocols [14] but that these should allow for some iterative adaptation, allowing for some secondary and exploratory analyses to be determined from interim analysis of emerging qualitative process evaluation data (stage 2, above). These additional analyses could be logged as updates on trial registers to pre-specify additional hypotheses that emerge during this period. This would support the utility and transparency of social science trials.

To help improve the reporting of realist RCTs, we also support the extension of the Consolidates Standards of Reporting Trials (CONSORT) statement for social and psychological interventions [36] which includes specific guidance for reporting on the intervention theory of change. We encourage that pre-hypothesised mediators and moderators also be included in reporting guidelines and that the post-evaluation empirically informed mid-range theory of change be reported. Proper reporting of theory and hypotheses by researchers may aid in applying research findings in practice. It would also allow systematic reviewers to integrate theory and process data when synthesising evidence on the effectiveness of interventions. Systematic reviewers aim to synthesise evidence on interventions with homogenous theories of change, but this is rarely reported sufficiently, leaving reviewers to fall back on synthesising interventions with vaguely similar components [15, 37–39].

Finally, further infrastructure investment is required to embed social science expertise within registered clinical trials units (CTUs) if they are to be a step change in the how trials of complex public health interventions are undertaken, moving from the medical model of investigating effectiveness towards a realist view which emphasises the importance of robust causal explanation. One possibility is that, in the long term, specialist Social Science Trial Units will also be accredited to support the design and conduct of social science trials that develop, test and refine mid-range programme theory via the three-stage process proposed.

## Conclusion

This paper provides guidance on the theoretical and methodological process of undertaking a realist RCT using the example of the evaluation of the Learning Together intervention. The main products of a realist RCT are both an evaluation of particular intervention effectiveness in a particular context as well as a nuanced mid-range theory of change, rather than outputs which merely accredit an intervention as effective or not. This paper is important in providing an example for researchers, practitioners and policymakers seeking a framework through which to develop and evaluate interventions which go beyond ‘what works’ and allows them to apply a *realist* approach.

## Abbreviations

CMO: context-mechanism-outcome configuration
CONSORT: Consolidates Standards of Reporting Trials
CTU: clinical trials unit
LT: Learning Together
MRC: Medical Research Council
NERS: National Exercise Referral Scheme
NIHR: National Institute for Health Research
RCT: randomised controlled trial
SES: socio-economic status
